# Supplementary material for: Imaging in-operando LiCoO2 nanocrystallites with Bragg coherent X-ray diffraction
Source: Commun Chem. 2024 Oct 27;7:243. doi: 10.1038/s42004-024-01331-y (PMC11514306; doi:10.1038/s42004-024-01331-y)
Supplement: Supplementary file 2 — Supplementary Material [file 42004_2024_1331_MOESM2_ESM.pdf]

Supplementary Material for Imaging in-operando $LiCoO_2$ Nanocrystallites with Bragg Coherent X-ray Diffraction

David Serban,[*,†,‡] Daniel G. Porter,[‡] Ahmed H. Mokhtar,[†] Mansoor Nellikkal,[†] Uthay Sivaperumal,[¶] Min Zhang,[§] Stephen P. Collins,[‡] Alessandro Bombardi,[‡] Peng Li,[‡] Christoph Rau,[‡] and Marcus C. Newton[*,†]

†*Department of Physics & Astronomy, University of Southampton, United Kingdom*
‡*Diamond Light Source, Harwell Oxford Campus, Didcot, United Kingdom*
¶*Department of Physics, Royal Holloway University of London, United Kingdom*
§*School of Chemistry, University of Southampton, United Kingdom*

E-mail: das1g13@soton.ac.uk; m.c.newton@soton.ac.uk

S1 Supplementary Method: Coin Cell Preparation

The sample under investigation consists of an $\approx 300\ \mu m$ layer of Kapton with a $2.2\ \mu m$ thick Al layer to ensure electric conductivity. The Kapton layer maintains rigidity, ensuring the inertness of the nanocrystals during the experiment. The Kapton-Al substrate then underwent cleaning using an ultrasonic bath containing two solutions: initially, acetone was used to remove any organic matter, followed by a cleaning solution of IPA.

LCO nanocrystals were applied via spin-coating to achieve a uniform layer of crystallites on the Al surface. The spin-coating process involved preparing a 200 ml isopropanol (IPA) solution containing approximately 1 mm^2 of powdered LCO. The substrate was placed in a spinning machine, programmed to spin it for 60 s at 200 rpm. During the spinning process, the LCO-IPA solution (thoroughly and constantly mixed) was dispensed onto the substrate in increments of 50 ml over approximately the first 30 s, using a micropipette.

The LCO powder utilised in the spin-coating procedure was obtained from a polycrystalline cylindrical LCO boule measuring $\approx$ 9 cm in length and 8 mm in thickness. This boule was grown in-house by the Department of Physics, Royal Holloway University of London, UK. The same boule underwent sectioning using a CO_2 laser to produce a smaller cylindrical boule suitable for mounting on the target holder of the Pulse Laser Deposition apparatus (Figure S.1.a). Following the cutting process, any debris was eliminated using a He gun and by rinsing in an IPA solution. Subsequently, the LCO target was affixed to the target holder, and further LCO was deposited onto the substrate via Pulse Laser Deposition (PLD) using a shadow mask to achieve regions of varying densities (Figure S.1.b).

An KrF excimer laser bombarded the solid LCO target with a beam of wavelength 248 nm and 20 mJ energy at room temperature (20°C) in high vacuum conditions ($< 10^{-4}$ mbar) during the PLD (Figure S.1.b). The pulse frequency of at least 100 Hz with a duty cycle of 10% created a plume of LCO that deposited on the Al surface. Higher than 200 Hz frequencies tended to form undesirable films of the material instead. The high density regions are useful to determine the predisposed alignment of crystals.

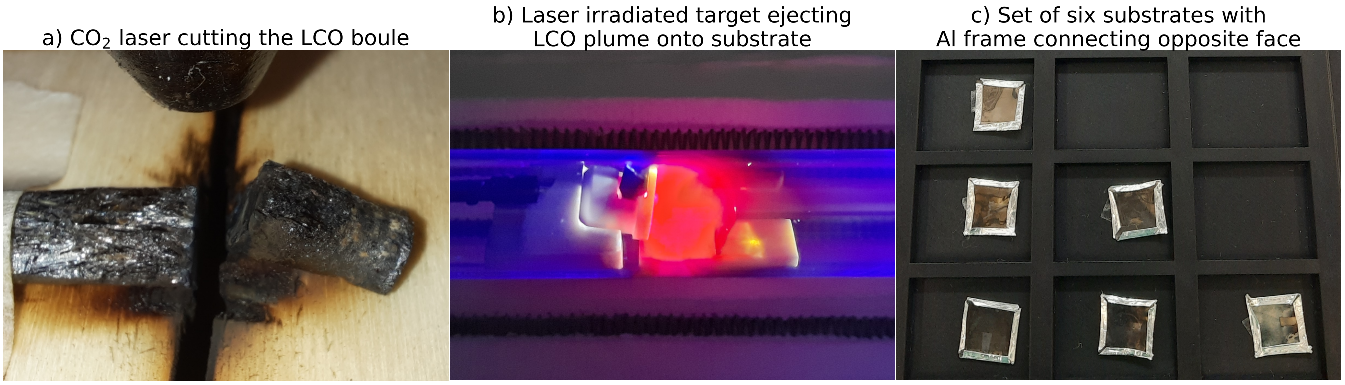

Figure S.1: Cathode preparation stages: a) CO_2 laser cutting of the larger boule to the smaller PLD target; b) PLD process of LCO boule irradiated by KrF excimer laser from the right pluming LCO plasma and condensing onto the prepared Al-Kapton substrate in a high-vacuum quartz tube; c) Final cathodes after spin-coating and PLDing LCO and framing with the Al tape to connect the opposite substrate's face to the conductive layer.

The lower density regions are exclusively for Bragg CDI irradiation to focus on single crystallites. Also, PLD ensured a degree of ablation that hindered the nanocrystals' movements under ionising beams and applied voltages.

The LCO nanocrystals were, therefore, located on a single side of the substrate - the conductive side. However, the substrate is meant to touch the holed steel cap on the Kapton side as the LCO must be submerged into the electrolyte to become a viable battery. Therefore, to ensure connection between the LCO on the Al side and the steel cap touching the Kapton side, an Al tape frame of similar dimensions to the substrate was positioned and bent both faces during coin cell assembly (Figure S.1.c).

Finally, the cathode was submerged in electrolyte along a fabricated graphite anode, while the two electrodes were separated by a Celgard 2400 monolayer microporous membrane. The formed assembly was clamped between a pair of CR2032 coin cell steel caps.

S2 Supplementary Discussion: Experiment Currents

Plots I, III, and IV of Figure S.3 particularly show a clear charging current, as the coin cell stabilises towards the end of the 5 minutes into an almost flat line. This is a typical and expected behaviour.

Plot II of Figure S.3 starts with a flat current until after more than 4 minutes it abruptly increases to a value close to the last ones measured during the previous state (Plot I of Figure S.3). Possibly, local crystallites only then begin to charge as the accumulated stimulation due to the higher voltage over time finally overtakes whatever resistance the crystallites might intrinsically have at lower voltages.

Plot V of Figure S.3 also has a negligibly null current for the first ≈ 30 s, until it peaks to a positive value. The shape of the graph suggests a slight charging state. Therefore, the coin cell loses charge faster than a commercial coin cell as it already has a very low capacity due to the small content of cathode material. In Plot V of Figure S.3, the coin cell is charged back to 3.5 V after slowly discharging during the previous stage Bragg CDI rocking curve scans, when it possibly discharged lower than the 3.5 V.

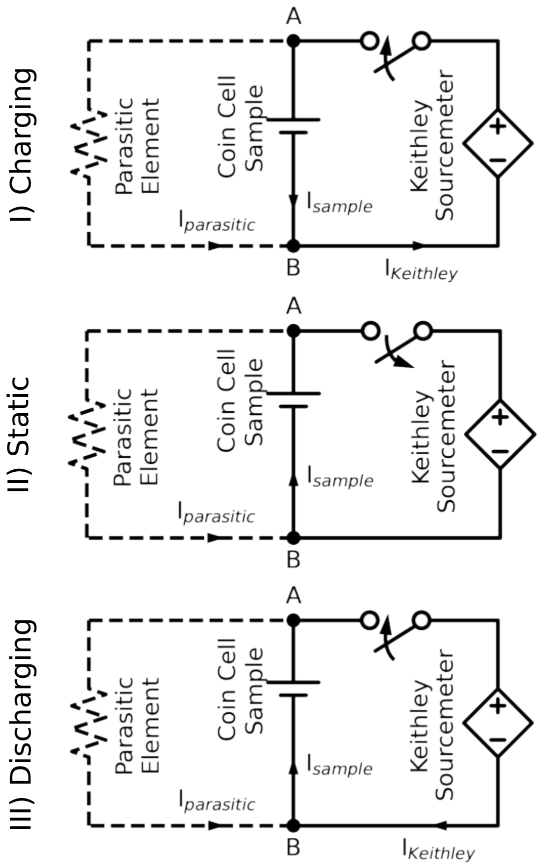

Figure S.2: Schematics displaying the different circuit conditions occurring during the experiment: I) when charging the coin cell, II) when the Keithley's power supply is interrupted during the Bragg CDI rocking curve scans, III) when discharging the coin cell. The Keithley Sourcemeter is a variable power source which is set throughout the experiment at different voltages to be applied to the coin cell sample. An added switch is drawn to assist the reader with the condition of the Keithley's circuit segment, but it is fully integrated into the sourcemeter. Furthermore, the Keithley device also measures the voltage across and the current through itself. The coin cell sample signifies the specifically designed coin cell for this experiment. The parasitic element and its respective circuit segment closes the circuit during the Bragg CDI rocking curve stages, however, its value and cause are somewhat unknown. Each of the currents passing through the circuit's segments has homonymous labels. This schematic is highly hypothesised based on our understanding, measurements, and other experiences with similar experimental setups.

Plot VI of Figure S.3 shows a similar behaviour to Plot V of Figure S.3; however, the general trend is more likely to resemble an actual discharge as the function's monotonic trend is increasing. The positive current is misleading, as it is clear that the coin cell is slowly discharging due to an unknown circuit-closing parasitic element that we discuss in more detail in the next paragraphs. Therefore, the measured current is positive and splits somewhere to supply the parasitic element, while drawing current from the coin cell as well. The lower the current from the coin cell runs (coin cell discharging), the higher the current from the Keithley power source rises.

Figure S.4 shows the same measured currents as all the subplots of Figure S.3, however, overlapped for a better understanding of the scale of each of the states. The graph of the currents when discharging with 3.0 V is insignificant compared to the ones when charging with 2.5 V, 3.5 V, and with $4.0V$, and when discharging with 3.5 V. Considering that these measurements are separated by approximately 7 h each - during which the rocking curve scans are capturing the diffraction patterns, assumed idle time - the battery most probably is draining considerably. The amount of charge being lost during the rocking curve scans depends on the immediately previous voltage applied (i.e., supplied stronger voltage right before the rocking curve scans implies stronger discharge during the following idle time).

Figure S.2 describes the circuit conditions during any of the stages. Figure S.2.I shows the charging stages, where the potential applied to the coin cell hypothetically generates a current through a parasitic circuit segment that is parallel to the sample. The current flowing through the sourcemeter is thus split into the main segment through the coin cell, and the parasitic segment. Considering the measured currents reaching an almost null equilibrium, we can deduce from this that it is possible that this parasitic element has an impedance much higher than the coin cell segment, as the current begins to be hindered through the coin cell when it is fully charged.

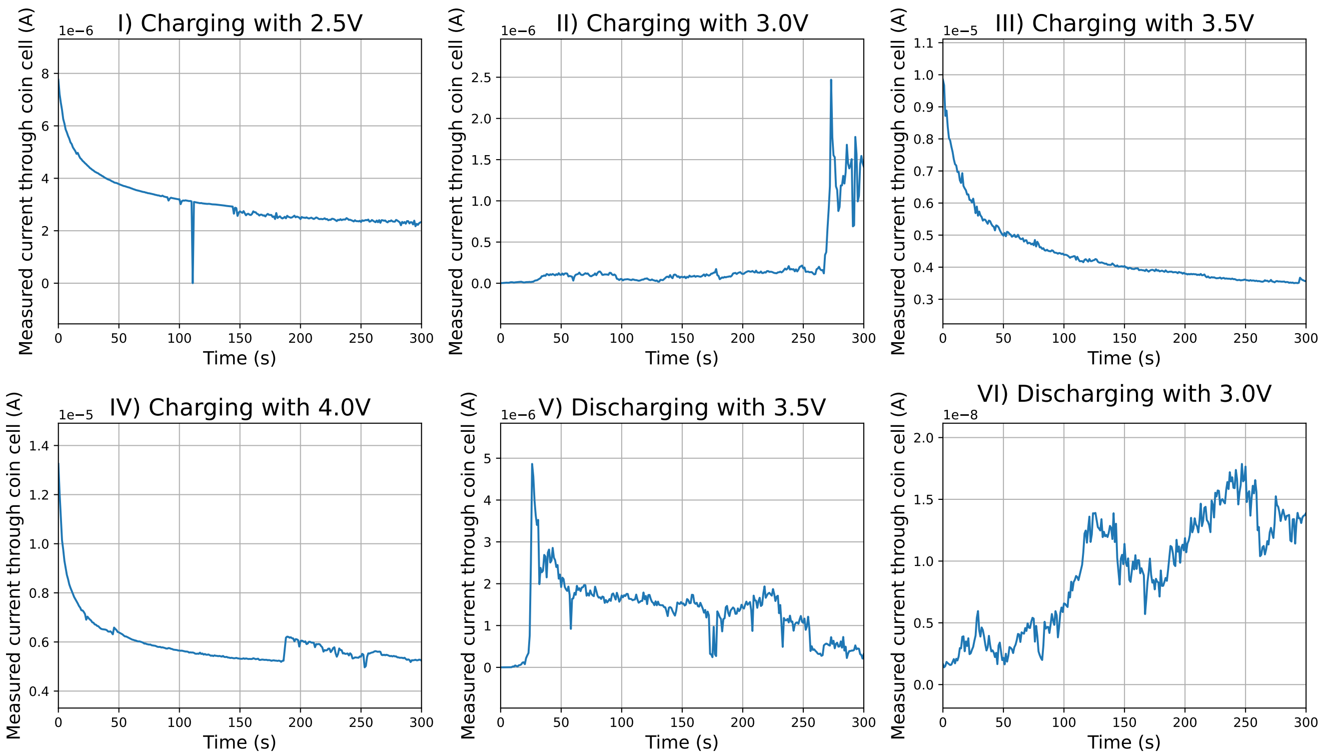

Figure S.3: Individual measured currents through the coin cell at the beginning of each cycling stage's series of Bragg CDI rocking curve scans to evidence the exponential behaviour.

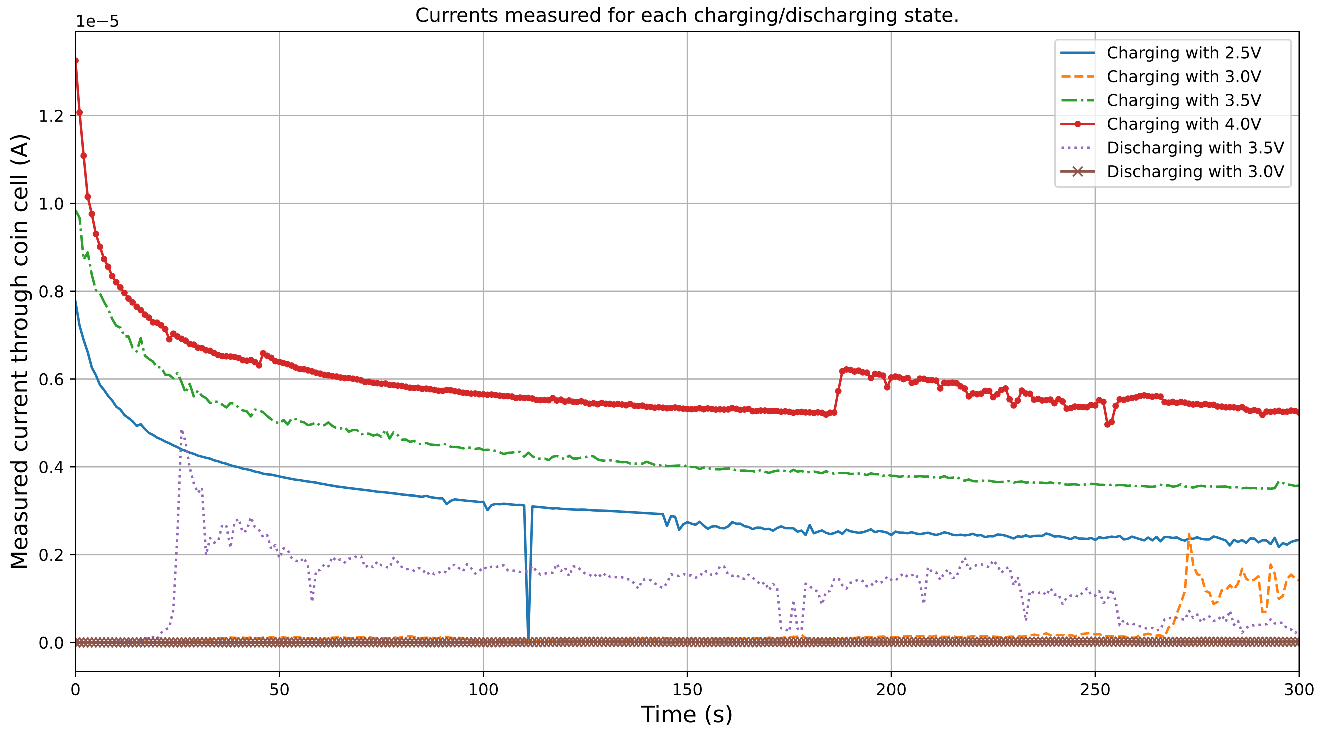

Figure S.4: Overlapped measured currents through the coin cell at the beginning of each cycling stage's series of Bragg CDI rocking curve scans for a better understanding of the currents' scale.

Figure S.2.II is the same circuit after the switch has been opened, occurring after every period of 5 minutes during which voltages are applied to the coin cell. Ideally this should be a static circuit condition, when no currents flow through any of the elements. However, our hypothesis is that the parasitic element is still connected to the coin cell, thus closing the circuit, and has a low-enough impedance to allow a discharging current to flow through. This circuit condition is achieved during the Bragg CDI rocking curve scans acquisition stage, when the sourcemeter is switched off (equivalent to the switch opening the circuit segment) and turned into a high-impedance mode.

Figure S.2.III is again the same circuit after sourcemeter is re-activated and its voltage is set to a lower voltage. This simulates a discharging protocol, where electrons begin to flow from the anode to bring the potentials to equilibrium states. In other words, the current through the coin cell and the sourcemeter should be reversed compared to the initial charging states.

The Figure S.2 explanation of the measurements observed in Figures S.3 and S.4 might be the reason why the currents are always positive and have a tendency to decrease with time even during the discharging voltages' application, as the discharge occurs slowly during the idle time spent performing rocking curves. This constant discharge through the parasitic element could be of different natures which contribute: X-ray beam ionising and discharging the coin cells slowly and/or high impedance circuit not being truly infinite impedance (within or outside the coin cell).

As another example from a similar experiment on an identical coin cell and by using the same experimental setup, we have eventually successfully charged the battery with 3.5 V and applied decreased voltages to observe the decaying exponential curve in smaller increments of voltages (0.1 V for three subsequent scans), and finally attempted to recharge the coin cell using 3.5 V again. Figure S.5 shows the measured currents for each of the scans overlapped as to suggest the asymptotic tendency of the currents in time. The chronologically first measurement (scan #1000251) is a positive direct current of $\approx$ 534 nA considered as the equilibrium value because it was achieved after charging the low-capacity coin cell for approximately 6 hours. Following this, we decreased the applied voltage in increments of 0.1 V to replicate a battery discharging protocol (scans #1000252, #1000253, and #1000254) and measured the currents following an exponential decay with a negative amplitude, asymptotic towards approximately the observed current in scan #1000251. After the three consecutive measurements showing expected discharge currents graphs, we attempted to recharge the battery with the 3.5 V to obtain a measurement of a charging stage. Due to the previous three scans removing charges from the anode every time, charging back with 3.5 V in scan #1000255 shows a stronger and more abrupt current evolution, which is possibly expected due to the relative voltage between scan #1000255 and #1000254, but also due to the capacity allowing stronger currents.

Therefore, all current measurements behave as expected, but with an offset apparently dependent on the applied voltage and the trend of the measurement's function. All the charging and discharging curves show an exponential tendency that were successfully fitted to the same functional defined in Eq. S.1 with different parameters distinguishing between the rate of currents change, the amplitude that clearly suggests if the battery is charging or discharging (as expected, negative if discharging, positive if charging).

$$i(t) = A\ b^{-\lambda(t-t_0)} + I_0 \tag{S.1}$$

However, the objective of Figure S.5 is to display the asymptotic value (I_0 of each curve fitting) being positive regardless if charging or discharging. In other words, this enforces the suspicion of the existence of the parasitic element branch in the circuit displayed in Figure S.2 that allows a continuous DC supply through the entire circuit, most probably even when the circuit is opened.

Furthermore, a function ignoring the I_0 con-

Table S.1: The parameters of the fitting curves for the respective scans displayed in Figure S.5. The amplitude (A) is consistent with the behaviour of the coin cell considering each respective voltage (negative if discharging, positive if charging). Similarly, the calculated charge (q) moved during each scan replicates the behaviour, as well as the calculated variation in Li ion population within the cathode. The decaying factors (λ) have the same order of magnitude and the offset DC (I_0) is always positive.

Scan #	Voltage (V)	A (nA)	b	λ (mHz)	t_0 (s)	I_0 (nA)	q (μC)	Li^+ (pmol)
1000252	3.4	-46.47	2.172	41.40	20	514.8	-2.69	27.92
1000253	3.3	-42.07	1.852	50.31	20	500.2	-2.45	25.43
1000254	3.2	-41.55	1.934	48.70	20	486.2	-2.40	24.91
1000255	3.5	100.7	3.558	50.22	20	549.8	5.65	-58.64

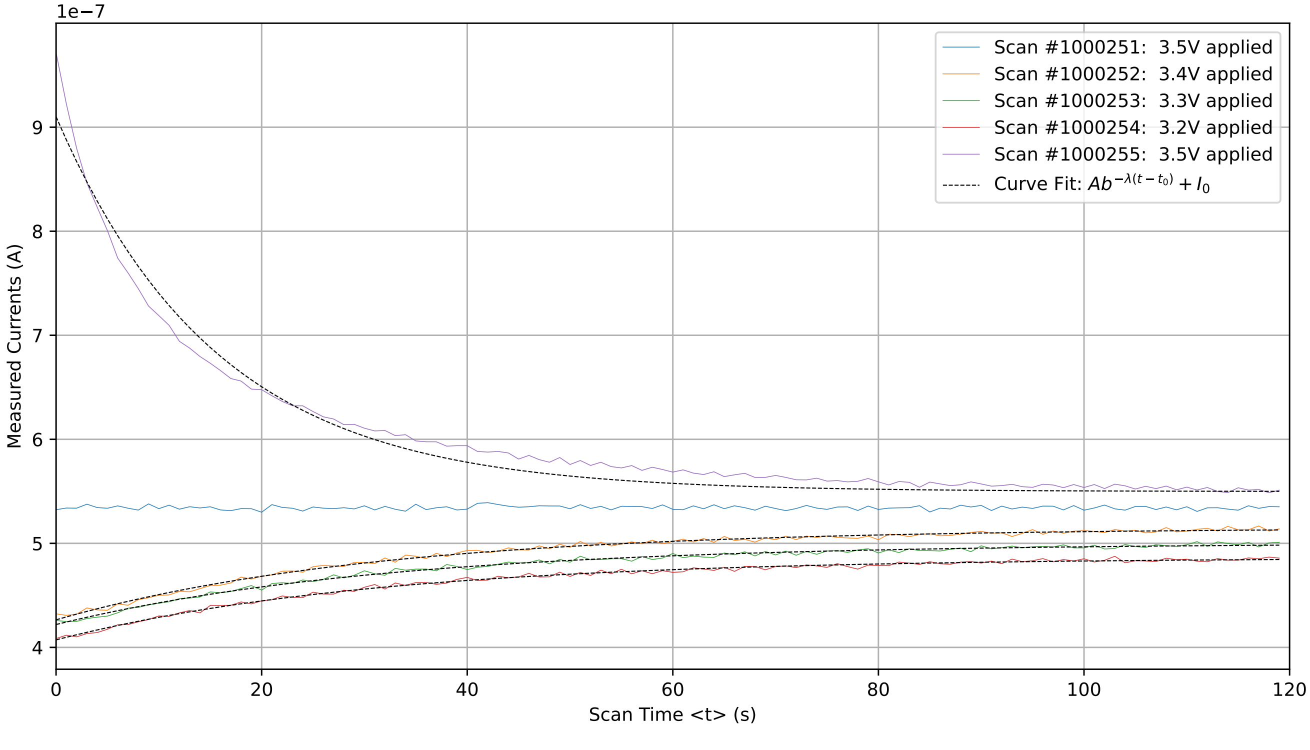

Figure S.5: Measured currents during an experiment when an identical coin cell was fully charged with 3.5 V (scan #1000251) and then partially discharged with three voltages: 3.4 V (scan #1000252), 3.3 V (scan #1000253), and 3.2 V (scan #1000254). Finally, the coin cell was charged with 3.5 V (scan #1000255) again. All scans display expected behaviours and fitting curves have sets of expected parameters determined and displayed in Table S.1. The direct current that is offsetting each measurement is always positive.

stant of each measurement should be truer to the discharging and charging currents through the coin cell. Therefore, integrating each function will return the approximate charge moved during each measurement.

$$\delta q = i(t)\delta t \implies q(t_f) = \int_{t=0}^{t_f} A\ b^{-\lambda(t-t_0)} dt = \\ = \frac{A\ b^{-\lambda(t-t_0)}}{-\lambda \log b}\Big|_{t=0}^{t_f} = \frac{i(t_f) - i(0)}{-\lambda \log b} \tag{S.2}$$

Table S.1 shows the determined parameters of the fitted exponential functional defined in S.1 to each of the discharging and charging scans in S.5 and on the last column the integrated value of the total charge moved in each separate scan. Of most significant note, the amplitude (A) values are as expected for each of the cycling scans, the decaying parameter (λ) values are of the same order of magnitude, the calculated charges moving in each measurement are of the correct sign associated with the charging and discharging currents (and of the same order of magnitude), and the offset current (I_0) is positive oscillating between 480 and 550 nA.

S3 Supplementary Notes: Peaks shift

Figures S.6 and S.7 show the calculated drifts of the centroid from the observed diffraction pattern in the detector plane in 2Θ and χ scattering angles, respectively. Figure S.6 also shows the calculated c-axis length of the crystal's primitive cell, by assuming the a and b unit cell lengths are constant. This calculation is explained in detail in Section S9.

A decrease in the 2Θ angle corresponds to an elongation of the c lattice parameter of the crystal lattice, and vice versa. Therefore, Figure S.6 shows a somewhat consistent behaviour of the crystal lattice when charging and discharging occurs as the lattice dilates in the c axis with Li being displaced from it[1]. The large error bars are due to a significant variation in the reflection's angle in the first two charging states, which most probably happens due to the slow discharge happening during the rocking curve scans. However, the line's trend can be mostly correlated with the applied voltages, while considering the state after charging with 3.5 V to be altered or an outlier at most. The hypothesised plastic deformation happening in the crystal also manifests in the lattice c parameter here, as the trend of the axis shortening starts with the 4 V state.

Figure S.7 shows the calculated χ angle of the peak and interestingly the trend can be correlated with the applied voltage. This typically suggests that the crystal is rotating, however, because the peak returns with the plastic deformation and the discharging voltages, we hypothesise that the scattering vector is tilted out of the initial reflection plane as higher voltages are applied. In other words, the Q-vector rotates in the bisector plane between the incident and the initial reflected beam.

Following this, as the crystal begins to be discharged, the scattering vector returns towards the initial position. These scattering vector dynamics can be associated with Li layers being more depleted on some sides of the crystal than on the others, generating strain and general displacements varying on the edges. Therefore, the CoO_2 layers can be separated locally more on some sides, facilitating the Q-vector movement and thus reflecting at slightly different χ angles.

We calculated the capacity of another reference coin cell in Section S10 that was prepared identically, but probably has a larger quantity of active LCO.

S4 Supplementary Method: Common Morphology

We define the set containing all the reconstructed states in Eq S.3, charging all the way up to 4.0 V in increments of 0.5 V and discharging with 3.5 V and 3.0 V.

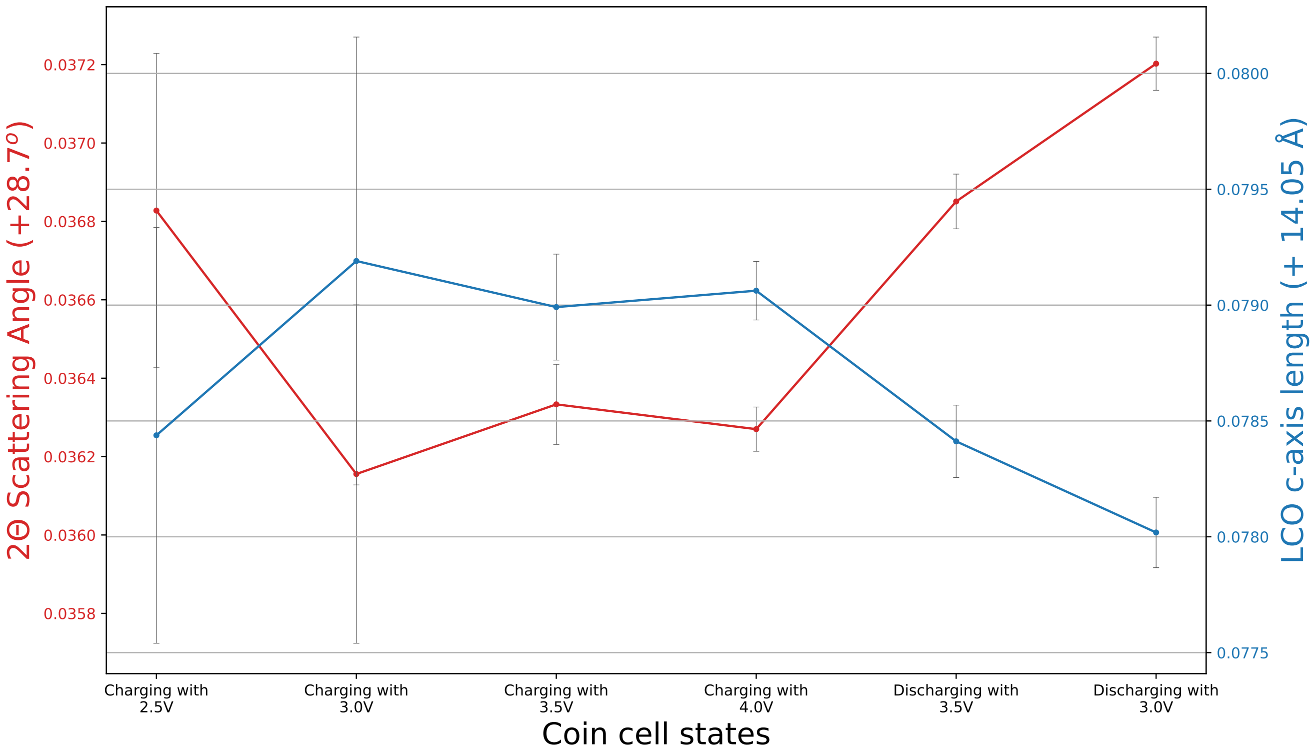

Figure S.6: The amount by which the peak's 2Θ angle shifts (red) and the variation in the primitve unit cell's c-axis length (blue) when cycling the battery, calculated from the centroid's position in the detector from each scan. The lengths of the error bars are the calculated standard errors of means.

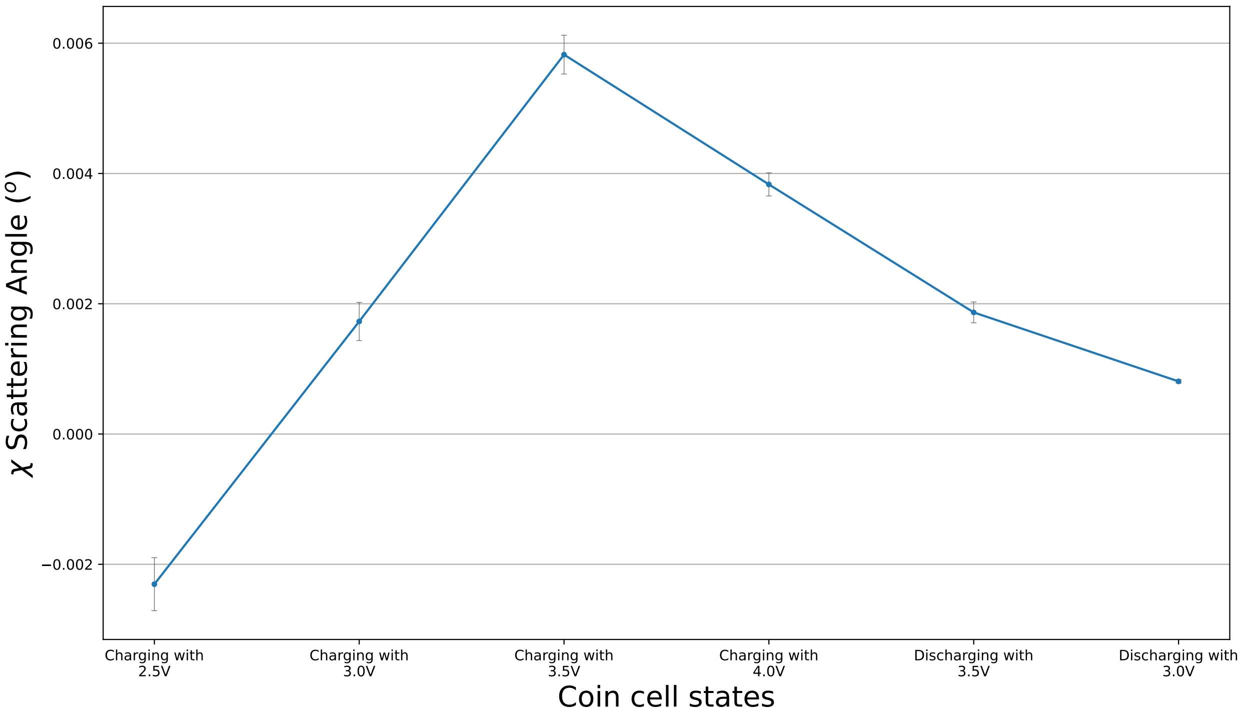

Figure S.7: The amount by which the peak's χ angle shifts when cycling the battery, calculated from the centroid's position in the detector from each scan, and calculated standard error of mean error bar lengths.

$$S = \{2.5\text{ V, Charging with 3.0V,} \\ \text{Charging with 3.5V, 4.0V,} \\ \text{Discharging with 3.5 V,} \\ \text{Discharging with 3.0 V}\} \tag{S.3}$$

We further note the reconstructions array of each of the state belonging to the set of complex numbers of their respective 3-dimensional shapes as in Eq. S.4.

$$R_s = \{r^s_{ijk} \in \mathbb{C} : \forall i \in \mathbb{N} \cap [1, m_s] \\ \wedge j \in \mathbb{N} \cap [1, n_s] \wedge \\ k \in \mathbb{N} \cap [1, p_s]\} \in \mathbb{C}^{m_s \times n_s \times p_s}, \forall s \in S \tag{S.4}$$

A further reasonable analysis requires comparable phase arrays. Therefore, a required common array shape results by obtaining the maximum values among all the three possible dimensions of the initial arrays (Eq. S.5),

$$\alpha = \max\{\alpha_s : \forall s \in S\}, \forall \alpha \in \{m, n, p\} \tag{S.5}$$

determining the padding values for each state along all three directions (Eq. S.6),

$$\delta^\alpha = \alpha - \alpha_s, \forall \alpha \in \{m, n, p\}, \forall s \in S \tag{S.6}$$

and equally padding the sides with null values to ensure the actual reconstructions are in the centre (Eq. S.7).

$$R^{`}_s = \left\{ \begin{array}{ll} r^{`}_{ijk} & = r_{i-\frac{\delta^m_s}{2}, j-\frac{\delta^n_s}{2}, k-\frac{\delta^p_s}{2}} \in R_s : \\ & i \in \left[\frac{\delta^m_s}{2}, \frac{\delta^m_s + m_s}{2}\right] \cap \mathbb{N} \wedge \\ & j \in \left[\frac{\delta^n_s}{2}, \frac{\delta^n_s + n_s}{2}\right] \cap \mathbb{N} \wedge \\ & k \in \left[\frac{\delta^p_s}{2}, \frac{\delta^p_s + p_s}{2}\right] \cap \mathbb{N} \\ r^{`}_{ijk} & = 0 : \text{otherwise} \end{array} \right\}, \\ \forall s \in S \tag{S.7}$$

Note that the machine learning algorithm requires the sizes of the arrays to be divisible by 16, therefore, the resulting reconstructions also have even number of voxels along any axis. Thus, when padding any of the smaller arrays to reach the shape of the largest reconstructions, the total number of padding voxels along any axis is even and, therefore, can be split in half and added at the ends of the original smaller array (i.e. $\frac{\delta^\alpha_s}{2}$, $\frac{\delta^\alpha_m + \alpha_s}{2} \in \mathbb{Z}$, $\forall \alpha \in \{m, n, p\}$, $\forall s \in S$)

Each of the elements within any of the reshaped arrays is a complex number, of which the phase is of the greater interest to the analysis. Consequently, arrays containing only the value of the phase found at each point share the same shape after padding (Eq. S.8).

$$\Phi_s = \left\{\arg\left(r^{`}_{ijk}\right) : \forall r^{`}_{ijk} \in R^{`}_s\right\} \in \mathbb{R}^{m \times n \times p} \tag{S.8}$$

S5 Supplementary Method: PCA

Since the phase arrays have the same shape, a subtraction operation between themselves can show the phase variations between any state a measurement chosen as a reference state. Considering the common shape, each phase array consists of an equal number of different real values, number which is determined by the product of the lengths along every direction (Eq. S.9).

$$N = mnp \tag{S.9}$$

Therefore, all the differences of phases arrays can be vectorised (Eq. S.10), i.e. reshaped into a vector of total length equal to the number of total real values within each of the phase arrays (Eq. S.11).

$$\begin{aligned} &\text{vec} : \mathbb{R}^{m\times n\times p} \rightarrow \mathbb{R}^N \\ &\text{vec}\,(X) = \{y_q \in \mathbb{R} : y_q = x_{ijk} \in X, \\ &\qquad\qquad q = np(i-1) + p(j-1) + k\} \end{aligned} \tag{S.10}$$

$$|\Phi_s\rangle = \text{vec}\,(\Phi_s) \in \mathbb{R}^N,\, \forall s \in S \tag{S.11}$$

Because the pristine stage measurement does not exist, the one obtained from charging with 2.5V is the chosen reference state relative to which the others are analysed, hereby noted as $|\Phi_0\rangle$ (Eq. S.12).

$$|\Delta\Phi_s\rangle = |\Phi_s\rangle - |\Phi_0\rangle \in \mathbb{R}^N, \forall s \in S \tag{S.12}$$

Performing an outer product between each relative state vector and itself results in a set of equally shaped quadratic matrices. Added together, they return a single quadratic covariant operator of rank N (Eq. S.13), which can be considered to have N eigenstates (Ψ_ν as the νth eigenstate) belonging to N eigenvalues (λ_ν as the νth eigenvalue; Eq. S.14).

$$\hat{A} = \sum_{s\in S} |\Delta\Phi_s\rangle\langle\Delta\Phi_s| \in \mathbb{R}^{N\times N} \tag{S.13}$$

$$\hat{A}|\Psi_\nu\rangle = \lambda_\nu|\Psi_\nu\rangle,\, \forall\nu \in [1, N] \cap \mathbb{N} \tag{S.14}$$

The eigenvector which has the largest statistical influence on the operator, in other words belonging to the largest eigenvalue is defined by the same index (Eq. S.15), and is called the principal component of the covariant system. It can be used in iterative dot products with the initial vectorised phase variation matrices to determine the component along this basis eigenvector of each of the phase differences. This results in a set of six values, one for each state, representing the reconstructed phase information along the principal component axis (Eq. S.16), reducing the number of variables while preserving as much information as possible.

$$\begin{aligned} \lambda_a = \max\{\lambda_\nu \in \mathbb{R} : \hat{A}|\Psi_\nu\rangle = \lambda_\nu|\Psi_\nu\rangle, \\ \forall\nu \in [1, N] \cap \mathbb{N}\} \end{aligned} \tag{S.15}$$

$$|\Omega\rangle = \{\langle\Delta\Phi_s \,|\, \Psi_a\rangle : \forall s \in S\} \in \mathbb{R}^{\text{card}(S)} \tag{S.16}$$

Furthermore, by conceptually using this eigenvector of largest statistical influence as a mask on each of the reconstructions, the determination of the regions that have the largest weight on the principal component analysis is feasible. To be computationally feasible, severe memory usage reduction is required - step fulfilled by binning the arrays (millions of voxels) every 4-by-4-by-4 (i.e. sum every square containing 64 voxels into one), and by masking out all the redundant null values after binning and then vectorising the result (Table S.2), though keeping a record of the positions of each of the points in the 3D array. The latter step is necessary to save time rather than anything else, as the immense number of null values would eventually return an equal number of null eigenvalues anyway, and thus, uninteresting.

The steps of binning and masking are easily reversible provided the array to be reversed is compatible with such operation, i.e. identical in shape with the arrays used to perform the PCA. However, the binning and null-values removal are not a pair of commutative operations.

To successfully display the relevant voxels and distinguish between all the phases within a state that influence the PCA and the ones that do not, each of the binned and masked relative phase arrays ($|\Delta\Phi_s\rangle$, $\forall s \in S$) and the maximum eigenstate itself ($|\Psi_a\rangle$, $\forall s \in S$) have their shapes restored (Eq. S.17) by filling in 3D ar-

Table S.2: Table showing numbers of 64 bit values found within array variables and the estimated occupied memory space by each array.

Memory reduction protocol	$\mathrm{card}(\lvert\Phi_s\rangle) = N$	$\mathrm{card}(\hat{A}) = N^2$
None	1 384 448 (10.6 MB)	1 916 696 264 704 (13.9 TB)
Binning 4-by-4-by-4	21 632 (165 KB)	467 943 424 (3.6 GB)
And after removing null values	78 (0.6 KB)	6 084 (46.4 KB)

rays of binned dimensions with the vectorised values recalling the previously filtered positions (Eq. S.17), or with null values the masked positions.

$$\begin{aligned} \mathrm{vec}_{m,n,p}^{-1} &: \mathbb{R}^N \to \mathbb{R}^{m\times n\times p} \\ \mathrm{vec}_{m,n,p}^{-1} &\circ \mathrm{vec} = \mathbf{1}_{\mathbb{R}^{m\times n\times p}} \end{aligned} \tag{S.17}$$

Consequently, the results should contain six (6) phase variation arrays along a seventh (7th) array deduced from the maximum eigenstate, all of the same dimensions as the initial phase arrays (Eq. S.18).

$$\begin{aligned} \Psi_a &= \mathrm{vec}_{m,n,p}^{-1}\left(\lvert\Psi_a\rangle\right) \\ \Delta\Phi_s &= \mathrm{vec}_{m,n,p}^{-1}\left(\lvert\Delta\Phi_s\rangle\right),\ \forall s \in S \end{aligned} \tag{S.18}$$

Then, a simple difference between each of the phase variation arrays and the maximum eigenstate results in a set of six (6) arrays of binned dimensions where the smaller values indicate good agreement with the analysis. The reciprocal of the absolute value at each set of coordinates is the optimal metric to proceed with since larger differences are prone to skew the analysis by showing crystal regions that do not influence the PCA outcome, and that the arrays contain real numbers (Eq. S.19).

$$\begin{aligned} \Delta_s = \Big\{ \tfrac{1}{\lvert \Delta\phi_{ijk}^s - \psi_{ijk}^a \rvert} \in \mathbb{R}\ :\ & \\ \Delta\Phi_s = \left\{\Delta\phi_{ijk}^s\right\} &\in \mathbb{R}^{m\times n\times p} \wedge \\ \Psi^a = \left\{\psi_{ijk}^a\right\} &\in \mathbb{R}^{m\times n\times p} \Big\},\ \forall s \in S \end{aligned} \tag{S.19}$$

S6 Supplementary Method: Q and G calculations

S6.i Q-vector calculation

We choose the laboratory frame of reference to be defined by the orthonormal basis of the incident X-ray beam in the positive z axis, the y axis towards the ceiling ($\hat{\mathbf{y}} = -\hat{\mathbf{g}^*}$, where $\hat{\mathbf{g}^*}$ is the unit vector of the apparent gravity), and the x-axis by the cross product of the y and z basis vectors ($\hat{\mathbf{x}} = \hat{\mathbf{y}} \times \hat{\mathbf{z}}$). Therefore, the incident beam's wavevector is defined as in Eq. S.20

$$\mathbf{k_{in}} = k\hat{\mathbf{z}} = \begin{pmatrix} 0 \\ 0 \\ k \end{pmatrix} \tag{S.20}$$

where $k = \frac{2\pi}{\lambda}$ and λ is the X-rays' wavelength.

Rotating any vector around the y axis by an angle of α is defined by the operator in Eq. S.21. Computationally, all the following calculations where simplified and possible after developing modular software. At the root of any rotation matrix, we applied the particular case on the general formula from Eq. 9.63 by Cole.[2] This modularisation was necessary once the different conventions of the planes and rotating axis became confusing.

$$\hat{R}_y(\alpha) = \begin{pmatrix} \cos\alpha & 0 & \sin\alpha \\ 0 & 1 & 0 \\ -\sin\alpha & 0 & \cos\alpha \end{pmatrix},\ \forall\alpha \tag{S.21}$$

The Bragg CDI rocking curve scans performed at i13 were tilting the sample around the vertical axis and reflecting the beam towards the

detector situated in the horizontal plane at an angle δ away from the 0th order. Therefore, considering the reflections were elastic, this is analogous to rotating the wavevector around the y-axis by the δ value, as in Eq. S.22.

$$\mathbf{k_{out}} = \hat{R}_y(\delta)\mathbf{k_{in}} = \begin{pmatrix} k\sin\delta \\ 0 \\ k\cos\delta \end{pmatrix} \tag{S.22}$$

Therefore, the scattering vector is the difference between the reflected and the incident wavevectors as in Eq. S.23.

$$\mathbf{Q} = \mathbf{k_{out}} - \mathbf{k_{in}} = \begin{pmatrix} k\sin\delta \\ 0 \\ k\left(\cos\delta - 1\right) \end{pmatrix} \tag{S.23}$$

And in the particular case of this experiment's laboratory frame of reference (δ=28.8^o, beam energy of 13.5 keV), Eq. S.24.

$$\begin{aligned} \mathbf{Q} &= \begin{pmatrix} 3.29 \\ 0 \\ -0.85 \end{pmatrix} \cdot 10^{10}\ \mathrm{m}^{-1} \\ \|\mathbf{Q}\| &= 3.398 \cdot 10^{10}\ \mathrm{m}^{-1} \end{aligned} \tag{S.24}$$

All the figures containing reconstructed objects have drawn axes that represent the laboratory frame of reference defined previously and used through the scattering vector calculation. Therefore, the calculated scattering vector has identical components in each of the frames, i.e. parallel to its unit vector defined in Eq. S.25.

$$\hat{\mathbf{Q}} = \frac{\mathbf{Q}}{\|Q\|} \approx \begin{pmatrix} 0.969 \\ 0 \\ -0.249 \end{pmatrix} \approx \begin{pmatrix} 4 \\ 0 \\ -1 \end{pmatrix} \tag{S.25}$$

S6.ii G-vector calculation

Table S.3 shows the lattice parameters of LCO (hexagonal) that were determined from an *in-house* X-ray Diffraction experiment:

Therefore, the primitive lattice vectors are defined as follows in Eq. S.26.

$$\begin{aligned} \vec{a_1} &= a \cdot \begin{pmatrix} \cos\frac{-\pi}{3} & \sin\frac{-\pi}{3} & 0 \end{pmatrix} \\ \vec{a_2} &= a \cdot \begin{pmatrix} \cos\frac{\pi}{3} & \sin\frac{\pi}{3} & 0 \end{pmatrix} \\ \vec{a_3} &= c \cdot \begin{pmatrix} 0 & 0 & 1 \end{pmatrix} \end{aligned} \tag{S.26}$$

Using the primitive lattice vectors as the rows within, we define the primitive lattice matrix in Eq. S.27.

$$\vec{a_i} \cdot \vec{a_j^*} = 2\pi\delta_{i,j} \implies \hat{A} = \begin{pmatrix} \frac{a}{2} & \frac{-a\sqrt{3}}{2} & 0 \\ \frac{a}{2} & \frac{a\sqrt{3}}{2} & 0 \\ 0 & 0 & c \end{pmatrix} \tag{S.27}$$

From which we can determine the reciprocal lattice vectors (Eq. S.30) as the columns from the reciprocal lattice matrix (Eq. S.29), which is the inverse of the primitive lattice matrix scaled by 2π.

$$\det\hat{A} = a^2c\left(\frac{\sqrt{3}}{4} + \frac{\sqrt{3}}{4}\right) = \frac{a^2c\sqrt{3}}{2} \implies \tag{S.28}$$

$$\begin{aligned} \hat{A}^{-1} &= \frac{4\pi}{a^2c\sqrt{3}} \begin{pmatrix} \frac{ac\sqrt{3}}{2} & \frac{ac\sqrt{3}}{2} & 0 \\ \frac{-ac}{2} & \frac{ac}{2} & 0 \\ 0 & 0 & \frac{a^2\sqrt{3}}{2} \end{pmatrix} = \\ &= \begin{pmatrix} \frac{2\pi}{a} & \frac{2\pi}{a} & 0 \\ \frac{-2\pi\sqrt{3}}{3a} & \frac{2\pi\sqrt{3}}{3a} & 0 \\ 0 & 0 & \frac{2\pi}{c} \end{pmatrix} \implies \end{aligned} \tag{S.29}$$

$$\begin{aligned} \vec{a_1^*} &= \begin{pmatrix} \frac{2\pi}{a} & \frac{-2\pi\sqrt{3}}{3a} & 0 \end{pmatrix} \\ \vec{a_2^*} &= \begin{pmatrix} \frac{2\pi}{a} & \frac{2\pi\sqrt{3}}{3a} & 0 \end{pmatrix} \\ \vec{a_3^*} &= \begin{pmatrix} 0 & 0 & \frac{2\pi}{c} \end{pmatrix} \end{aligned} \tag{S.30}$$

Therefore, the general lattice effect vector depending on the Miller indices can be defined as in Eq. S.31.

Table S.3: LiCoO3 Lattice parameters. Pinsard-Gaudart et al.[3] touch on the subject of the lattice parameters differing due to different crystal growth conditions. Therefore, since the nanocrystal could either be from the spin-coating process or the PLD process, we cannot surely state the parameters, but we can use the approximate values to continue this calculation.

a (Å)	b (Å)	c (Å)	$\alpha(^o)$	$\beta(^o)$	$\gamma(^o)$
2.834	2.834	14.054	90	90	120

$$\begin{aligned}\mathbf{G}(k,h,l) =& h\vec{a_1^*} + k\vec{a_2^*} + l\vec{a_3^*} = \\ =& 2\pi \begin{pmatrix} \frac{(h+k)}{a} & \frac{\sqrt{3}(k-h)}{3a} & \frac{l}{c} \end{pmatrix}\end{aligned} \quad \text{(S.31)}$$

Considering the particular parameters of the lattice from Table S.3 and the experimental setup for the $(1, 0, -5)$ reflection, we obtain the lattice effect vector for our crystal in Eq. S.32.

$$\begin{aligned}\mathbf{G}(1,0,\text{-}5) =& \begin{pmatrix} 2.21 \\ \text{-}1.28 \\ \text{-}2.24 \end{pmatrix} \cdot 10^{10}\ \text{m}^{-1} \\ \|\mathbf{G}\| =& 3.395 \cdot 10^{10}\ \text{m}^{-1}\end{aligned} \quad \text{(S.32)}$$

The determined scattering vector (Eq. S.24 and lattice effect vector (Eq. S.32) have magnitudes that differ by less than 0.1%; therefore, we can conclude from these calculations that the deviation is insignificant enough to be convinced of the crystal being LCO and its reflection being truly the $(1, 0, -5)$. The variation could be due to the imprecision or approximations when the in-house X-ray diffraction was performed, or during the actual *in-operando* Bragg CDI rocking curve acquisitions.

Despite the magnitude, the orientations of the components are completely different (the angle between the Q-vector and the G-vector is approximately 37.2^o), but this is expected, considering that the orientation of the crystal does not necessarily have to be in any way parallel to the laboratory frame of reference. Therefore, because the orientation of the crystal lattice cannot be determined from a single scattering vector, there is no point in attempting to align the lattice effect vector with the scattering vector.

S6.iii Strain calculation

Because of the impossibility of determining the lattice orientation from a single reflection, the atomic displacements within the reconstructions cannot be determine unless as the components along the scattering vector. Therefore, choosing and focusing on the scattering vector as the single axis currently, we know that the phase is therefore just the product between the magnitude of the **Q**-vector and the magnitude of the local atomic displacement along the **Q**-vector (Eq. S.33).

$$\phi = \mathbf{Q} \cdot \mathbf{u} \implies \phi = \|\mathbf{Q}\|\, u_{\mathbf{Q}} \quad \text{(S.33)}$$

where $u_{\mathbf{Q}}$ is the atomic displacement along the scattering vector.

From the previously calculated scattering vector values (Eq. S.24) and the spectrum of phase between $-\pi$ and π, we can therefore determine the spectrum of atomic displacement along the scattering vector using Eq. S.34.

$$u_{\mathbf{Q}} = \frac{\phi}{\|\mathbf{Q}\|} \in [-0.924, 0.924]\ \text{Å} \quad \text{(S.34)}$$

And because the crystal orientation is uncertain, strains along the primitive lattice vectors are also uncertain. However, by adapting the strain tensor's definition to the scattering vector as a principal axis, we can represent the strain along the scattering vector instead. Considering that the Q-vector is almost parallel to the x-axis, we approximate the strain along the scattering vector to be similar to the one along the x-axis (Eq. S.35).

$$\epsilon_{ij} = \frac{1}{2}\left(\frac{\partial \mathbf{u}_i}{\partial x_j} + \frac{\partial \mathbf{u}_j}{\partial x_i}\right) \implies \epsilon_{\mathbf{Q}} \approx \frac{\partial u_{\mathbf{Q}}}{\partial x} \quad \text{(S.35)}$$

The real-space laboratory frame of reference visualisations contain discreet coordinates that were measured using Bonsu, from which we could measure the resolution of the reconstructions to be of approximately 49.5 nm in the x-axis. From this, we can state that the strain tensor's covariant along the scattering vector is approximately between −0.00372 and 0.00372 (Eq. S.36).

$$\epsilon_{\mathbf{Q}} \approx \frac{\Delta u_{\mathbf{Q}}}{\Delta x} \in [-0.00372, 0.00372] \tag{S.36}$$

S7 Supplementary Note: Multiple slices along the three axis of the reconstructions

Figure S.8 shows additional slices of the reconstructed objects along all three axes at different points along the crystal.

S8 Supplementary Note: Slices through the centroids of the diffraction patterns and the Fourier Transforms of the reconstructions

Figure S.9 shows slices taken of the diffraction patterns obtained during the experiment, after being masked at the lowest threshold possible to exclude random noise in the detectors. Figure S.9 also contains slices of the Fourier Transform of the reconstructions respective of the diffraction patterns found above each. All the slices are taken along one of the three array axes, but all through the centroid of the respective diffraction pattern.

The same arrays that are shown in Figure S.9 were used in the χ-squared calculations. Therefore, the main manuscript contains the calculated differences between observed and calculated diffraction patterns, while here we have decided to display some of the visual differences. Though the Fourier Transform of the reconstructions generally look blurry and less scattered compared to the observed diffraction patterns, commonly strong features are observable, and, therefore, holistically conclusively similar.

S9 Supplementary Method: Lattice Parameter Calculation

Determining any primitive lattice length of a crystal requires knowing the scattering angle of a reflection, the respective Miller indices of the reflection, the X-ray wavelength, and the other lengths of the primitive lattice.

First, applying Bragg's law to determine the magnitude of the scattering vector:

$$Q = \frac{4\pi}{\lambda} \sin \frac{2\Theta}{2} \tag{S.37}$$

where Q is the magnitude of the scattering vector, λ is the wavelength of the X-rays, and 2Θ is the scattering angle.

The scattering vector's magnitude can also be determined by performing the scalar product between itself and the scattering vector's conjugate:

$$Q^2 = \langle Q \,|\, Q \rangle \tag{S.38}$$

We can extend any calculation like the following to multiple dimensions, not just in a 3D space. Therefore, proceeding with a general N-dimensional discussion will allow our particular 3D space to be correct while also confirming any other. To this end, we define a set of orthonormal N-dimensional basis:

$$E_n = \{|e_1\rangle, |e_2\rangle, \ldots |e_n\rangle\} \tag{S.39}$$

and every primitive/reciprocal lattice vector is completely defined using the basis from Eq.

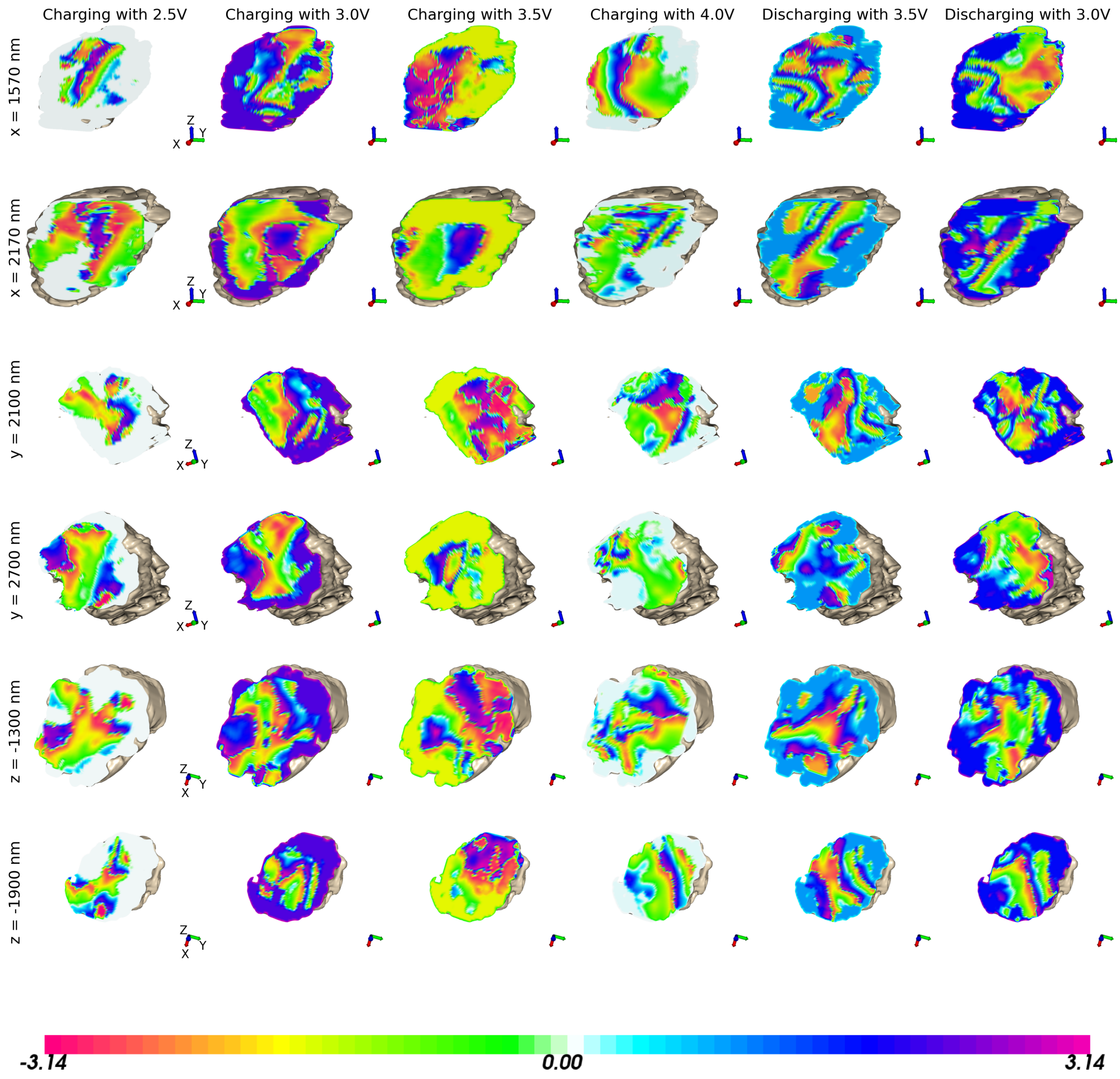

Figure S.8: Slices through the reconstructions at different coordinates along all the axes. The rectilinear axes system is in the laboratory frame of reference. N.B. The slices from the main manuscript were taken of all the reconstructions across the x-axis to intersect the approximate centre of mass of the reconstructions (x ≈ 1870 nm), as the x-axis was the axis along which slices would be most exposed from a view point on the scattering vector's direction and most significant and diverse phase changes would be visible (i.e. surface vs inner crystal phase changes).

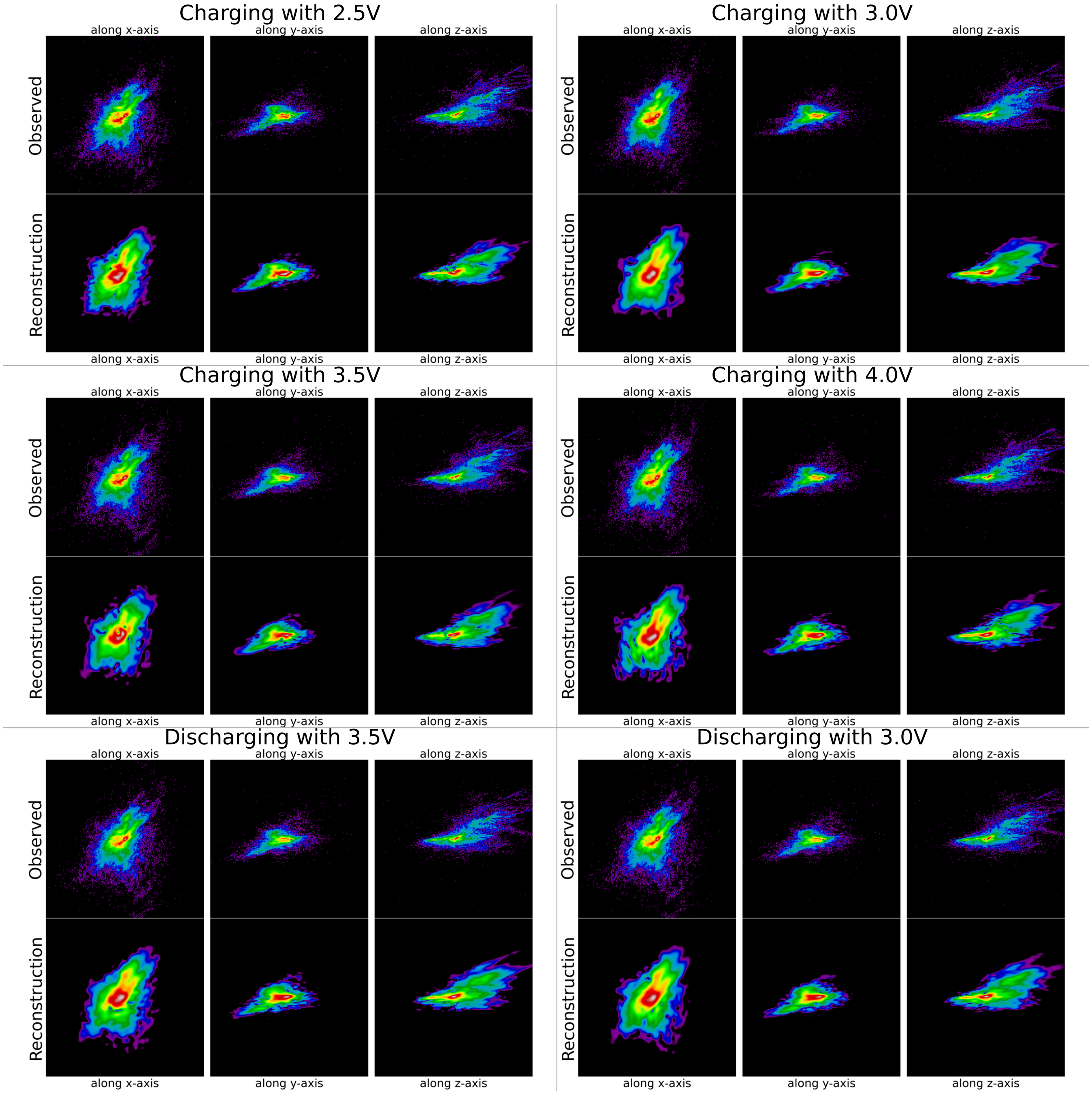

Figure S.9: Slices through the three array axes (x, y, z from left to right) of the experimentally observed diffraction pattern (above) and the calculated Fourier Transform of the resulting reconstruction (below) for each of the six coin cell cycling stage.

S.39:

$$\langle a_i \mid e_j \rangle = a_{i,j} \qquad \langle b_i \mid e_j \rangle = b_{i,j} \tag{S.40}$$

where $a_{i,j}$ and $b_{i,j}$ are the components of the ith primitive lattice vector ($|a_i\rangle$) and the ith reciprocal lattice vector ($|b_i\rangle$), respectively, along the jth basis vector:

$$|a_i\rangle = \sum_j a_{i,j} |e_j\rangle \tag{S.41}$$

$$|b_i\rangle = \sum_j b_{i,j} |e_j\rangle \tag{S.42}$$

Therefore, we can define the scattering vector as a superposition of the reciprocal lattice vectors with the Miller indices as weights:

$$|Q\rangle = \sum_i h_i |b_i\rangle \tag{S.43}$$

where h_i is the ith Miller index and $|b_i\rangle$ is the ith reciprocal lattice vector.

From Eq. S.38 and S.43:

$$Q^2 = \left(\sum_i \langle b_i | h_i \right) \left(\sum_i h_i |b_i\rangle \right) \tag{S.44}$$

and, by extending further:

$$Q^2 = \sum_{i,j} h_i h_j \langle b_i \mid b_j \rangle \tag{S.45}$$

We can further consider each of the reciprocal lattice vectors as the product between its magnitude and its unit vector:

$$|b_i\rangle = b_i \; |\hat{b_i}\rangle \tag{S.46}$$

Therefore, the scalar product between two reciprocal lattice vectors found within Eq. S.45 can be further split into the product between the same vectors' magnitudes and the scalar product between their unit vectors.

$$Q^2 = \sum_{i,j} h_i h_j b_i b_j \langle \hat{b_i} \mid \hat{b_j} \rangle \tag{S.47}$$

The scalar product between two reciprocal lattice vectors' unit vectors is subunitary and can simply be replaced with the cosine of the angle between the two:

$$\langle b_i \mid b_j \rangle = \cos \beta_{i,j} \tag{S.48}$$

where $\beta_{i,j}$ is the angle between the ith and the jth reciprocal lattice vectors. Therefore, Eq. S.47 becomes:

$$Q^2 = \sum_{i,j} h_i h_j b_i b_j \cos \beta_{i,j} \tag{S.49}$$

The right-hand side of Eq. S.49 can further be split as a sum of quadratic terms (for when $i = j$) and a sum of products of alternatively different terms (for when $i \neq j$). We assume the unknown of this equation is the length of the xth reciprocal lattice vector. The other reciprocal lattice vectors, every Miller index, as well as x^{th} reciprocal lattice vector's direction are all known variables. Implicitly, all the angles between the reciprocal lattice vectors are also known. Therefore, Eq. S.49 is equivalent to a quadratic equation:

$$A \; {b_x}^2 + B \; b_x + C = 0, \text{ where} \tag{S.50}$$

$$A = h_x^2 \cos \beta_{x,x} \tag{S.51}$$

$$B = h_x \sum_{i \neq x} h_i b_i \left(\cos \beta_{i,x} + \cos \beta_{x,i} \right) \tag{S.52}$$

$$C = -Q^2 + \sum_{i,j \neq x} h_i h_j b_i b_j \cos \beta_{i,j} \tag{S.53}$$

The angle between any vector and itself is null.

$$\beta_{i,i} = 0, \forall i \implies \cos \beta_{i,i} = 1, \forall i \tag{S.54}$$

Therefore, Eq. S.51 can be further reduced to:

$$A = h_x^2 \tag{S.55}$$

The angle between a vector $|u\rangle$ and another vector $|v\rangle$ is opposite to the angle between the same vector $|v\rangle$ and the same vector $|u\rangle$ due to their reversal.

$$\beta_{i,j} = -\beta_{j,i} \implies \cos \beta_{i,j} = \cos \beta_{j,i}, \forall i, j \tag{S.56}$$

Therefore, the coefficient of the 1_{st} power in the

quadratic Eq. S.50 can be simplified as:

$$B = 2h_x \sum_{i \neq x} h_i b_i \cos \beta_{i,x} \tag{S.57}$$

Regarding Eq. S.53, we can further split the summation term by the cases when the indices are identical and when they are different, becoming:

$$\begin{aligned} C = & - Q^2 + \sum_{i \neq x} h_i^2 b_i^2 \cos \beta_{i,i} + \\ & + \sum_{i \neq j \neq x \neq i} h_i h_j b_i b_j \cos \beta_{i,j} \end{aligned} \tag{S.58}$$

Here we can apply the observations from Eqs. S.54 and S.56 to reduce further:

$$\begin{aligned} C = & - Q^2 + \sum_{i \neq x} h_i^2 b_i^2 + \\ & + 2 \sum_{i,j \neq x, j > i} h_i h_j b_i b_j \cos \beta_{i,j} \end{aligned} \tag{S.59}$$

Applying the numerical values for each of the lattice parameters in turn for any case will result in a set of coefficients for a solvable quadratic equation. The algorithm to solving a quadratic equation is well known and we will refrain from detailing it here. However, the signs of the factors, implicitly of the terms, and of the conditional solution suggest that the only real solution to the equation must be when adding the square root of the discriminant.

Finally, the single solution is the length of one reciprocal lattice vector. The reciprocal lattice is conventionally defined by:

$$\langle a_i \,|\, b_j \rangle = 2\pi \delta_{i,j} \tag{S.60}$$

where $\delta_{i,j}$ is the Kronecker delta and $\langle a_i|$ is the conjugate of the primitive lattice vector corresponding to the reciprocal $|b_i\rangle$ lattice vector.

Defining the angle between the primitive lattice and the reciprocal lattice unit vectors as γ, we can determine the length of the corresponding primitive lattice vector as below:

$$\cos \gamma_i = \langle \hat{a_i} \,|\, \hat{b_i} \rangle \implies a_i = \frac{2\pi}{b_i \cos \gamma_i} \tag{S.61}$$

The objective of the calculations above was to assist with writing any formulae for computational determinations of any (abstract or real) crystalline systems. We will continue to describe our particular case.

LCO application

The LCO lattice is hexagonal, therefore, $b_0 = b_1$, $\beta_{0,2} = \beta_{1,2} = \frac{\pi}{2}$, and $\beta_{0,1} = \frac{\pi}{3}$. Furthermore, in our experiment we have only looked at the $(1, 0, \text{-}5)$ reflection and it is more likely that throughout our measurements the c-axis is the only one that changed and needs to be determined from the magnitude of the scattering vector changing with the scattering angle 2Θ. Thus, Eqs. S.55, S.57, and S.59 become:

$$A = h_2^2 = (-5)^2 = 25 \tag{S.62}$$

$$B = 2h_2 \sum_{i=0}^{1} h_i b_i \cos \frac{\pi}{2} = 0 \tag{S.63}$$

$$\begin{aligned} C = & - Q^2 + \sum_{i=0}^{1} h_i^2 b_i^2 + 2h_0 h_1 b_0 b_1 \cos \frac{\pi}{3} = \\ = & - Q^2 + b_0^2 \end{aligned} \tag{S.64}$$

Solving the quadratic equation for the reciprocal c-axis length using the coefficients from Eqs. S.62, S.63, and S.64, we obtain:

$$b_2 = \frac{\sqrt{Q^2 - b_0^2}}{|h_2|} \tag{S.65}$$

Replacing the scattering vector magnitude from Eq. S.37, and each of the reciprocal lattice vectors' lengths from Eq. S.61, we obtain a final equation for the c-axis length:

$$a_2 = \frac{\lambda |h_2| a_0 \cos \gamma_0}{\cos \gamma_2 \sqrt{4a_0^2 \cos^2 \gamma_0 \sin^2 \frac{2\Theta}{2} - \lambda^2}} \tag{S.66}$$

where h_2=-5 is the third Miller index, a_0=2.834 Å is the first primitive lattice length assumed constant, $\gamma_0 = \frac{\pi}{6}$ is the angle between the first primitive lattice vector and the first reciprocal lattice vector, and $\gamma_2 = 0$ is the angle between the third primitive lattice vector and the third

reciprocal lattice vector.

S10 Supplementary Discussion: Reference Cell Capacity Calculations

Figure S.10 shows an SEM image of the surface of the window substrate with the general distribution of LCO and a magnification on a crystallite that has the approximate dimensions of the particle that we successfully reconstructed from the experiment.

Using Figure S.10, we have calculated the LCO covered area to be 44.3% on average. From Figure S.1.c, we approximate that the general substrate surface that was in contact with the electrolyte during our experiment to be a square of 7 mm lengths. Furthermore, we approximate that the average height of the nanocrystals on the surface as 5 μm. Therefore, the overall volume of LCO substance during the experiment that is in contact with the electrolyte should be $\approx 1.08 \cdot 10^{-4}$ cm^3. By using the LCO density of 5.08 g/cm^3, we obtain the cathode mass of $\approx$0.5505 mg.

We have determined the capacity of a reference coin cell that was manufactured using the same protocol as the coin cell we experimented on, except for a possible increase in LCO quantity. This difference should not affect the outcome of our measurements of the specific capacity of the coin cell. To be exact, the outcome is that the reference cell has a much lower specific capacity than typical coin cells. Therefore, our Bragg CDI experiment coin cell also has a much lower specific capacity than typical coin cells, even if the LCO quantity is lower than that of the reference coin cell. For our following calculations, we have considered the same LCO quantity as deduced from the SEM image (Figure S.10).

Figure S.11 shows measurements performed on the reference coin cell at the same voltages as during the synchrotron experiment, for comparison. The measurements when applying the initial discharging voltages are absolute, but, in fact, in the other direction relative to the charging measurements. Comparing the measurements of this reference coin cell (Figure S.11) with the ones of the other identical coin cells from Bragg CDI experiments (Figures S.3 and S.5), we can be certain that the Bragg CDI coin cells were successfully cycled.

To calculate the specific capacity, the protocol was as follows. We charged the reference coin cell at a constant voltage of 4.4 V and considered the reference coin cell charged once the measured current dropped below 0.3 mA (Figure S.12). Then, to discharge the same reference coin cell, a constant drain current was applied until the voltage reduced to 3.2 V. Figure S.13 shows the calculated specific capacities when the reference coin cell is discharged. The curves from Figure S.13 can be compared to the works of Meng et al.[4] (particularly Fig 3) to show that, as expected, our reference coin cell has a much lower specific capacity than typical. Note that probably this reference coin cell contains more LCO than the one from our experiment.

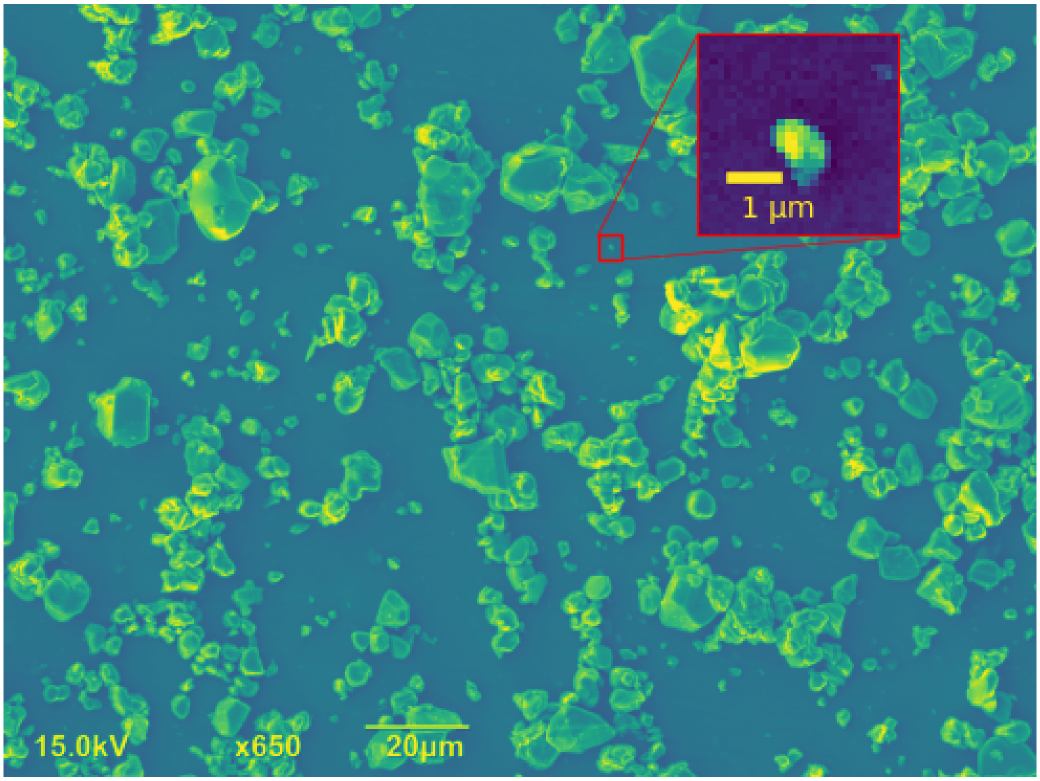

Figure S.10: SEM image of a region on the surface of the window substrate showing LCO cathode nanocrystals of different shapes and sizes. Magnification of a smaller region on a smaller nanocrystal that has approximately the same dimensions as the successfully reconstructed nanocrystal from our experiment.

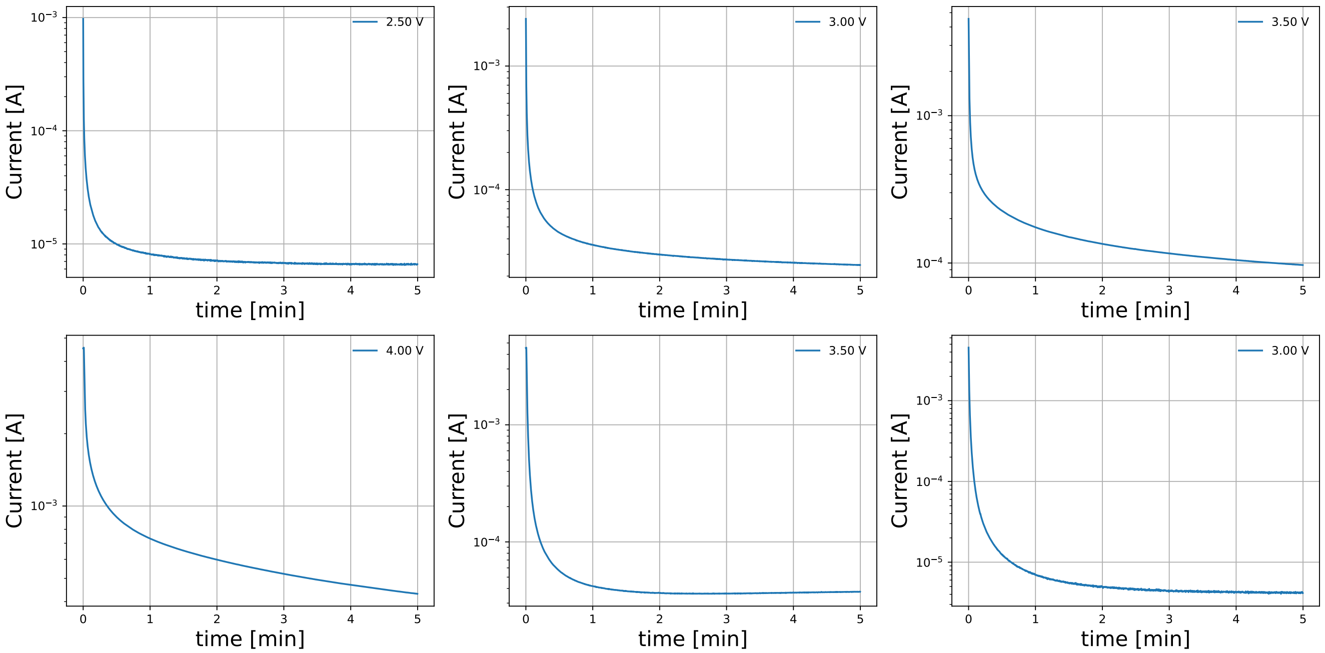

Figure S.11: Current measurements when the same fixed voltages from our Bragg CDI experiment are applied to charge and discharge the reference coin cell. The reference coin cell can contain more LCO than the coin cell from our Bragg CDI experiment.

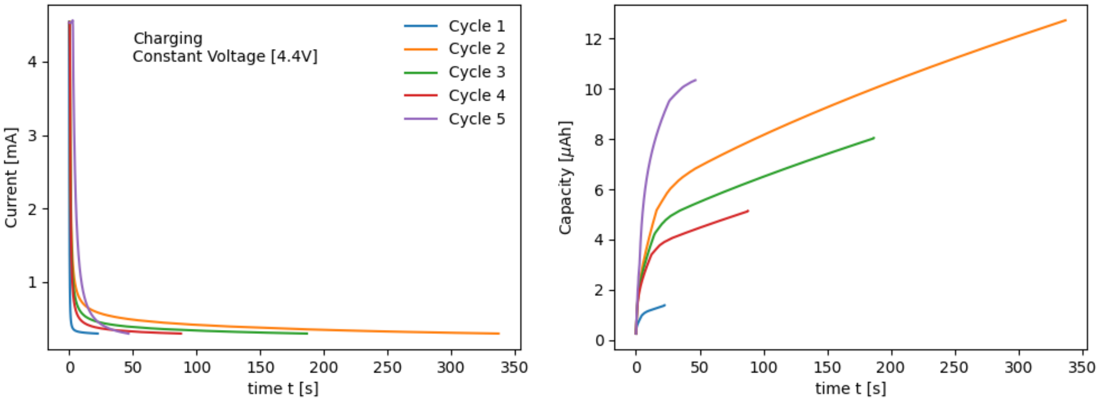

Figure S.12: Current vs time and Capacity vs time measurements during the charging stages of five consecutive cycles of our reference coin cell.

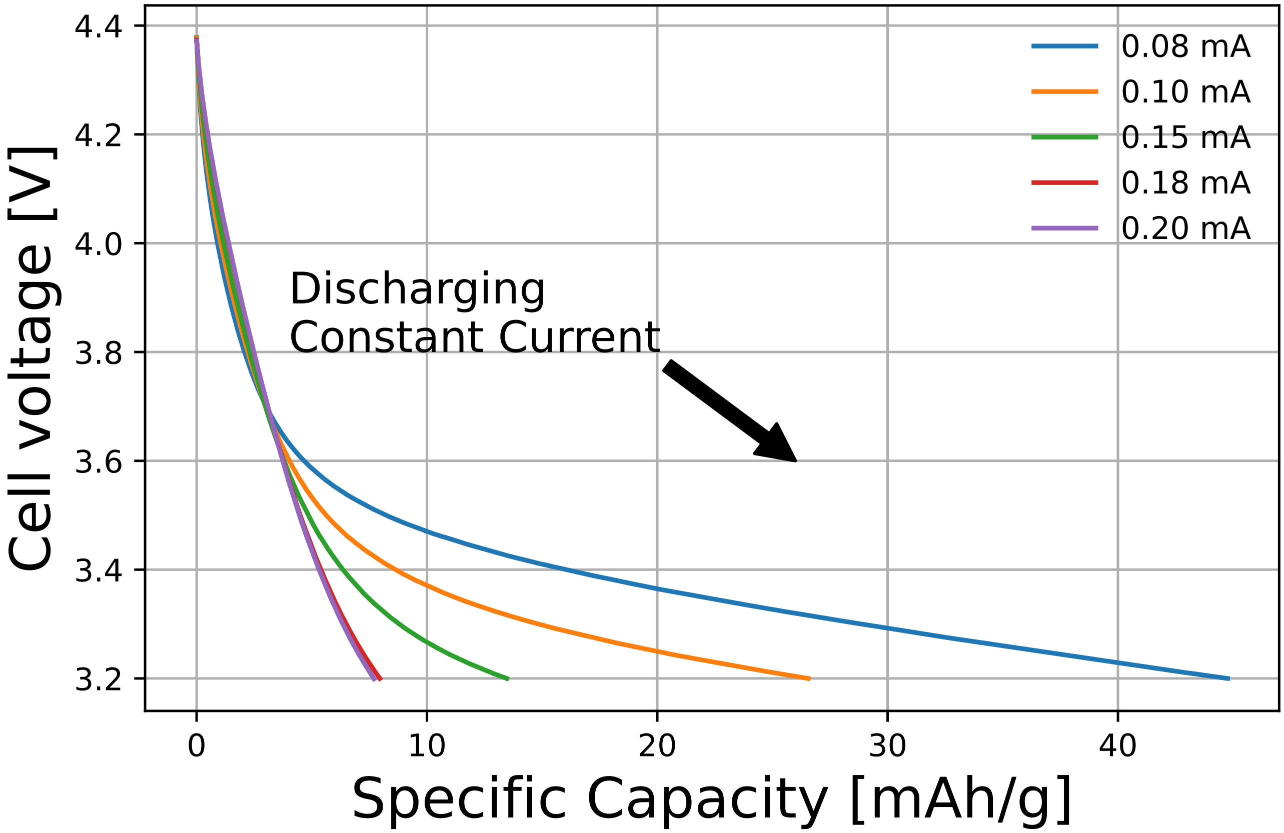

Figure S.13: Specific Capacity - voltage curves during the discharging cycles of the reference coin cell which can have more LCO than the Bragg CDI experiment coin cell. Compared to the measurements of Meng et al.,[4] these show a much lower specific capacity than typical attributed to our reference coin cell.

Supplementary References

(1) Estandarte, A.; Diao, J.; Llewellyn, A.; Jnawali, A.; Heenan, T.; Daemi, S.; Bailey, J.; Cipiccia, S.; Batey, D.; Shi, X.; Rau, C.; Brett, D.; Jervis, R.; Robinson, I.; Shearing, P. Operando Bragg Coherent Diffraction Imaging of LiNi0.8Mn0.1Co0.1O2 Primary Particles within Commercially Printed NMC811 Electrode Sheets. *ACS Nano* **2021**, *15*, 1321–1330.

(2) Cole, I. R. Modelling CPV. **2015**,

(3) Pinsard-Gaudart, L.; Ciomaga, V.-C.; Dragos, O.; Guillot, R.; Dragoe, N. Growth and characterisation of LixCoO2 single crystals. *Journal of Crystal Growth* **2011**, *334*, 165–169.

(4) Meng, Q.; Wu, H.; Mao, L.; Yuan, H.; Ahmad, A.; Wei, Z. Combining Electrode Flexibility and Wave-Like Device Architecture for Highly Flexible Li-Ion Batteries. *Advanced Materials Technologies* **2017**, *2*, 1700032–n/a.
