# Supplementary material for: Imaging in-operando LiCoO2 nanocrystallites with Bragg coherent X-ray diffraction
Source: Commun Chem. 2024 Oct 27;7:243. doi: 10.1038/s42004-024-01331-y (PMC11514306; doi:10.1038/s42004-024-01331-y)
Supplement: Supplementary file 3 — Description of Additional Supplementary Files [file 42004_2024_1331_MOESM3_ESM.pdf]

Description of Additional Supplementary Files

File name: Supplementary Movie 1

Description: Slices taken of the reconstructions showing phases along the X-axis.

File name: Supplementary Movie 2

Description: Slices taken of the reconstructions showing phases along the Y-axis.

File name: Supplementary Movie 3

Description: Slices taken of the reconstructions showing phases along the Z-axis.
